# WMSA–WBS: Efficient Wave Multi-Head Self-Attention with Wavelet Bottleneck

**DOI:** 10.3390/s25165046

**Published:** 2025-08-14

**Authors:** Xiangyang Li, Yafeng Li, Pan Fan, Xueya Zhang

**Affiliations:** School of Computer, Baoji University of Arts and Science, Baoji 721016, China; lixiangyang@stu.bjwlxy.edu.cn (X.L.); fanpan@bjwlxy.edu.cn (P.F.); zhangxueya@bjwlxy.edu.cn (X.Z.)

**Keywords:** vision transformer, self-attention, waveflow, wavelet transform, convolution neural network

## Abstract

The critical component of the vision transformer (ViT) architecture is multi-head self-attention (MSA), which enables the encoding of long-range dependencies and heterogeneous interactions. However, MSA has two significant limitations: its limited ability to capture local features and its high computational costs. To address these challenges, this paper proposes an integrated multi-head self-attention approach with a bottleneck enhancement structure, named WMSA–WBS, which mitigates the aforementioned shortcomings of conventional MSA. Different from existing wavelet-enhanced ViT variants that mainly focus on the isolated wavelet decomposition in the attention layer, WMSA–WBS introduces a co-design of wavelet-based frequency processing and bottleneck optimization, achieving more efficient and comprehensive feature learning. Within WMSA–WBS, the proposed wavelet multi-head self-attention (WMSA) approach is combined with a novel wavelet bottleneck structure to capture both global and local information across the spatial, frequency, and channel domains. Specifically, this module achieves these capabilities while maintaining low computational complexity and memory consumption. Extensive experiments demonstrate that ViT models equipped with WMSA–WBS achieve superior trade-offs between accuracy and model complexity across various vision tasks, including image classification, object detection, and semantic segmentation.

## 1. Introduction

Over the past few years, vision transformer (ViT) [1] has been widely adopted in a variety of computer vision tasks, such as image classification [2,3,4], object detection [5,6,7], and semantic segmentation [8,9,10]. ViT partitions an image into patches and encodes their features, leveraging the transformer’s multi-head self-attention (MSA) mechanism to model global relationships within the patch sequence for various computer vision tasks. Although the MSA mechanism enhances the model’s ability to handle global dependencies, certain specific tasks impose special requirements on MSA. For example, in industrial anomaly detection, capturing fine-grained features requires the ViT to process local detail information while reducing the computational burden. However, there are two challenges regarding ViT. Firstly, a primary challenge in employing MSA mechanisms is their inherent tendency to overlook crucial local details like edges, textures, and fine-grained features. Although effective at capturing global contextual relationships, they often struggle to extract important local features. A prevalent approach involves the integration of convolutional neural networks (CNNs) with MSA, offering a solution. For example, Duan et al. [11] proposed DUCT, which integrates dynamic unary convolution with multi-head self-attention in parallel. However, this approach also introduces a certain computational burden. Secondly, another challenge in applying self-attention is the quadratic computational cost that scales with respect to the number of input tokens. This computational complexity becomes particularly significant when dealing with large-scale or high-resolution images, leading to a substantial computational burden and memory requirements. To address the above problems, many researchers have attempted to design more efficient self-attention mechanisms [12,13,14,15]. Notably, PVT [13] reduces the number of keys and values through average pooling operations, thus reducing the computational burden. This method inevitably results in information loss as a consequence of the pooling operations they employ. MViT [15] performs pooling operations on queries, keys, and values, respectively, to reduce the computational burden. Although effective, these methods inevitably result in information loss as a consequence of the pooling operations they employ.

Our motivation is to design a simple yet effective MSA mechanism that reduces computational burden while enhancing the ability to capture local contextual information. We propose a novel wave bottleneck structure (WBS) that integrates the discrete wavelet transform (DWT) into the traditional bottleneck structure of ResNet. Meanwhile, we introduce wave multi-head self-attention (WMSA). We integrate the designs of WMSA and WBS into a unified module, referred to as WMSA–WBS. WMSA–WBS not only integrates the DWT characteristics from the wave bottleneck but also combines the strengths of convolution operations and self-attention mechanisms to capture both global and local information in images while maintaining efficiency. Notably, WBS integrates the bottleneck block with both 1D DWT and 2D DWT to reduce the number of parameters while preserving the network’s accuracy. WBS implements convolutional operations in the wavelet domain, thereby expanding the receptive field and enhancing the local contextual modeling capability. In WMSA, the spatial dimensions are reduced to one-fourth after the wavelet transform, which proportionally decreases the number of parameters in the self-attention mechanism. The input keys and values incorporate low-frequency information while also leveraging high-frequency information. The wave fusion module combines high-frequency and low-frequency information to provide diverse frequency-domain features as input for key/value operations. We further present an end-to-end model named WMSA–WBS vision transformer (WMSA–WBS–ViT) for visual representation learning. WMSA–WBS achieves a superior trade-off between accuracy and model complexity across diverse vision tasks, including image classification, object detection, and semantic segmentation. The integration of WMSA–WBS within WMSA–WBS–ViT results in notable improvements over the existing baseline methods. Our contributions are summarized as follows:

1. WBS: A wave bottleneck structure that integrates ResNet’s bottleneck block with DWT, aiming to reduce the number of parameters while maintaining the existing accuracy of the network.
2. WMSA: We introduce a novel WMSA module designed to mitigate computational cost. Within this module, the strategy of extracting and fusing different frequency information enables the model to better emphasize low-frequency semantics and high-frequency details.
3. WMSA–WBS: This module combines WBS and WMSA, and we design a WMSA–WBS–ViT as the backbone for experimentation.
4. Extensive Experiments: We perform extensive experiments across several datasets to evaluate the performance of WMSA–WBS–ViT. Specifically, we conduct image classification on the ImageNet-1K dataset, semantic segmentation on ADE20K, and object detection on COCO. Our approach is also compared with advanced baseline methods. The results showcase the superior performance of WMSA–WBS–ViT.

The following paper is organized as follows. Section 2 presents a review of the prior studies on the integration of wavelets with deep learning, along with advancements in the bottleneck structure and multi-head self-attention (MSA). Section 3 provides a detailed technical description of WBS, WMSA–WBS, and WMSA–WBS–ViT. In Section 4, we conduct a comprehensive evaluation of the proposed model across three computer vision tasks, with an in-depth analysis of its performance through ablation studies. Finally, Section 5 concludes the paper by summarizing our contributions and discussing potential application scenarios.

## 2. Related Work

### 2.1. Wavelets

A wavelet, known as a “mathematical microscope”, is a powerful mathematical tool capable of decomposing signals or images into information at different scales and selecting important information as needed. DWT decomposes data into various frequency components, enabling us to analyze each at a resolution suited to its scale. The advantages are as follows: (1) DWT is inherently reversible, guaranteeing that no information is lost when utilized specifically for downsampling purposes. (2) DWT effectively captures high-frequency information in images, aiding in the restoration of detailed features. (3) Using DWT can reduce image resolution, thus reducing memory consumption. Meanwhile, this process will not produce redundant parameters and can speed up the inference speed of the model, which benefits efficient model building. (4) DWT enhances the receptive field, allowing the model to capture more detailed features.

Many researchers have concentrated on integrating wavelets with deep learning [16,17,18,19]. It is widely applied in tasks such as noise-robust image classification [20], image restoration [21], image denoising [17], image segmentation [16], and medical image analysis [22]. Wavelets are integral to architectural design, significantly enhancing both CNN [23,24,25] and vision transformer models [14,26]. Wang et al. [23] introduce a CNN model that directly learns in the wavelet domain by utilizing wavelet transform during image preprocessing to extract information from high-resolution inputs. Li et al. [24] propose an efficient wavelet transformer (EWT) that employs DWT for downsampling to reduce image resolution while retaining important features and introduces multi-level feature aggregation and dual-flow feature extraction modules to effectively balance model performance and resource consumption. We [25] propose CasWTM to address the problem of traditional CNNs’ pooling operations potentially overlooking features that are crucial for classification. Wavelet vision transformer (Wave-ViT) [14] combines wavelet transforms with self-attention learning to achieve reversible downsampling, preserving image details while reducing computational costs. WaveFormer [26] is a novel approach that combines wavelet transform with a transformer architecture, aiming to address the issue of information loss inherent in the traditional methods.

### 2.2. Bottleneck

The bottleneck is first proposed in ResNet [27], aiming to solve the degradation problem in deep neural networks. With the growing demand for higher performance, researchers [28,29,30,31] have started exploring ways to improve ResNet’s bottleneck structure to enhance the model’s performance and efficiency. Gao et al. [32] propose Res2Net, which enhances the traditional ResNet bottleneck module by incorporating multi-scale feature representation, allowing each residual block to extract features at various scales and improving the model’s representational capacity. In [33], ResNeXt extends ResNet by introducing grouped convolution within bottleneck blocks, enhancing the model’s representational ability and parallel computation capability while maintaining manageable computational complexity. Gao et al. [34] propose BoTNet, which enhances traditional CNNs by integrating self-attention mechanisms into ResNet’s bottleneck module, thereby improving the model’s ability to capture global features and complex details.

### 2.3. MSA

In recent years, the improvements of MSA have mainly focused on two aspects: reducing computational complexity and improving overall MSA performance. Firstly, reducing the dimensionality of key and value inputs can effectively reduce computational demands. Specifically, Wang et al. [12] propose PVT v2, which uses AvgPool to reduce the spatial resolution of keys and values, significantly decreasing the computational burden. Wu et al. [35] propose P2T by using pyramid pooling to reduce the tokens of keys and values. Unfortunately, these approaches can inadvertently lead to loss of crucial information during the transformation process. Sparse attention is another effective method to lower complexity by skipping the calculation of unimportant attention weights, thus avoiding unnecessary resource usage. Beltagy et al. [36] introduce Longformer, which adopts a mixed local and global sparse attention mechanism. It calculates most of the attention weights within local regions while applying global attention to a few tokens, reducing the complexity to a linear level. Agent attention [37] is designed to optimize the trade-off between computational efficiency and representation capability. The acquisition of agent tokens is facilitated through a pooling process that still discards information. Although several methods have been proposed to simplify the computation of standard softmax self-attention, such as sparse attention and low-rank approximation, these approaches frequently result in reduced accuracy and limited speedup. Secondly, MSA performance can be improved based on specific task requirements. In medical image segmentation, Reza Azad et al. [22] propose incorporating boundary attention into the self-attention mechanism to enhance the capture of high-frequency information. For Face Super-Resolution, Li et al. [38] introduce RSA, a data-driven method that adaptively applies texture-aware reconstruction using a coarse-to-fine approach. Yao et al. [14] introduce Wave-ViT, which leverages wavelet transforms to downsample key and value inputs, thereby reducing computational cost. However, their design primarily focuses on spatial reduction and overlooks the rich structural cues encoded in high-frequency components, as well as the potential benefits of 1D wavelet-domain feature extraction. Our previous research [25] has demonstrated that high-frequency information is crucial for capturing local features. Notably, two of the aforementioned studies have leveraged high-frequency information, whereas Wave-ViT [14] has not fully exploited its potential. In contrast, our work enhances MSA’s capacity for capturing local features from three perspectives while ensuring its ability to capture global features. In wavelet-domain convolution, convolution operations are incorporated in the wavelet transform domain to efficiently extract local features. In joint modeling in 1D and 2D wavelet domains, convolution operations are applied separately in both 1D and 2D wavelet domains, enabling hierarchical processing of different frequency components. This approach not only enhances local feature extraction but also reduces computational complexity. Regarding the fusion of high-frequency and low-frequency information, we propose a method that fully exploits both high-frequency and low-frequency information to improve local feature capture. Our approach not only addresses the limitations of the existing methods in utilizing high-frequency components but also ensures the complementary integration of different frequency features, thereby providing MSA with a more powerful capability for local feature modeling.

Unlike previous studies, our work leverages wavelet transforms to simultaneously improve the global–local feature representation capacity of MSA and reduce its computational overhead. In this work, we propose WBS and WMSA. By leveraging the excellent properties of wavelets and combining the designed WBS and WMSA modules, WMSA–WBS not only reduces computational complexity but also better captures global features and local detail information in the spatial, frequency, and channel domains. Section 3 offers an in-depth discussion.

## 3. Our Method

In this section, we first review the components of the bottleneck in ResNet and multi-head self-attention (MSA) in ViT. We then present a detailed analysis of the technical design and advantages of the WBS and WMSA–WBS modules within the proposed WMSA–WBS–ViT network.

### 3.1. Preliminaries

**Figure 1:**
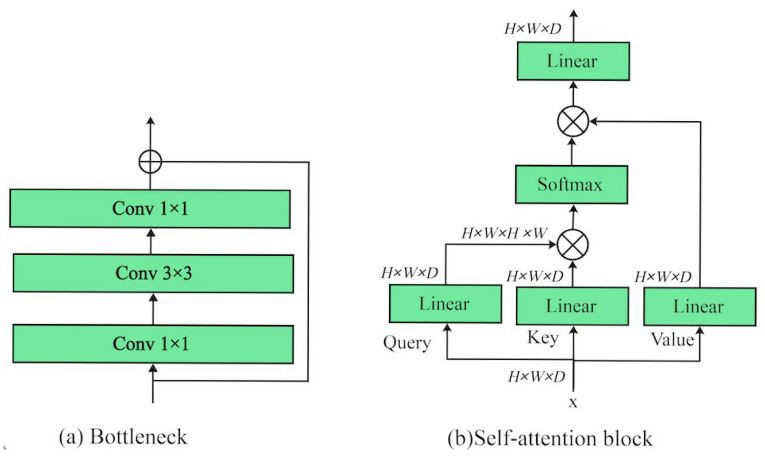
(**a**) Basic bottleneck block in ResNet; (**b**) basic self-attention block in ViT backbones.

**Bottleneck in ResNet.** As shown in Figure 1a, the traditional bottleneck architecture consists of three convolutional layers (conv 1 × 1, conv 3 × 3, and conv 1 × 1) designed to reduce dimensionality, extract spatial features, and restore the original channel size efficiently, enabling deeper networks with fewer parameters. Specifically, let $X\in R^{H\times W\times D}$ be the input image feature, where *H*, *W*, and *D* represent the height, width, and number of channels, respectively. For each residual block, a 3-layer stack is used. The input *X* first goes through a conv 1 × 1 for dimensionality reduction, followed by a conv 3 × 3 for feature extraction, and finally a conv 1 × 1 for dimensionality restoration. The residual connection adds the output to the input.

**Multi-Head Self-Attention in ViT.** As shown in Figure 1b, MSA uses multiple independent attention heads to process input data in parallel, with each head responsible for different subsets of features, calculating attention weights separately, and then concatenating their results to comprehensively capture diverse characteristics of the input. Specifically, given an input feature $X\in R^{H\times W\times D}$ representing the input patch sequence, where *H*, *W*, and *D* denote the height, width, and number of channels, respectively, the input is first reshaped into a sequence of N=H×W tokens: $X\in R^{N\times D}$. Three different linear layers are then used to generate the query $Q\in R^{N\times D}$, key $K\in R^{N\times D}$, and value $V\in R^{N\times D}$ matrices. The multi-head self-attention (**multi-head**) module splits each query/key/value into $N_{h}$ heads along the channel dimension, producing $Q_{j}$, $K_{j}$, and $V_{j}\in R^{N\times D_{h}}$ for the *j*-th head, where $D_{h}=D/N_{h}$. The self-attention (**attention**) mechanism computes the dot product between the query and key, scales it by $\sqrt{D_{h}}$, and applies a softmax function to obtain the attention weights. These weights are then used to compute the weighted sum over the value vectors, yielding the attention output for each head. All head outputs are concatenated and projected through a final linear layer to obtain the final attention output. Here, we show the general formula for classical MSA as follows:

(1) $$\begin{array}{cc} \; & h_{j}=\mathrm{Attention}\left(Q_{j},K_{j},V_{j}\right),\\ \; & \mathrm{Attention}\left(Q_{j},K_{j},V_{j}\right)=\mathrm{Softmax}\left(\frac{Q_{j}K_{j}^{⊤}}{\sqrt{D_{h}}}\right)V_{j},\\ \; & \mathrm{MultiHead}\left(Q,K,V\right)=\mathrm{Concat}\left(h_{1},h_{2},\ldots ,h_{n}\right)W. \end{array}$$

### 3.2. WMSA–WBS

**Figure 2:**
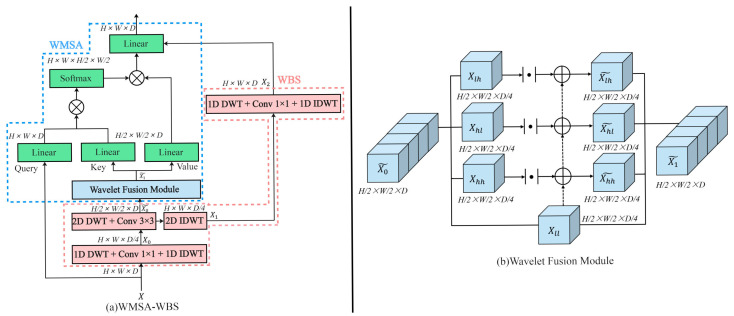
(**a**) Our proposed WMSA–WBS; (**b**) wave feature fusion module based on DWT.

We propose **WMSA–WBS**, a hybrid architecture that integrates wavelet-based multi-resolution analysis into a multi-head self-attention framework, aiming to enhance global–local feature representation under constrained computational budgets. As shown in Figure 2a, WMSA–WBS consists of two core components: the **wavelet bottleneck structure (WBS)** for compact local-context encoding and the **wavelet-enhanced multi-head self-attention (WMSA)** module for frequency-aware attention.

This design is inspired by the complementary strengths of wavelet transforms and self-attention. While MSA captures long-range dependencies, it lacks strong locality bias. Wavelet transforms, in contrast, offer low-cost multi-scale decomposition with localized spatial support. WMSA–WBS combines both for efficient and expressive representation learning.

**WBS** injects frequency-aware inductive bias into the backbone while preserving the spatial and channel resolutions. It consists of three consecutive wavelet-based processing stages that progressively extract, compress, and reconstruct informative representations in both spatial and frequency domains.

Given the input feature map $X\in R^{H\times W\times D}$, we first apply a 1D discrete wavelet transform (DWT) along a spatial axis to decompose the signal into directional low-frequency and high-frequency components. The transformed representation is then compressed by a group convolution block μ(·) and reconstructed using 1D Inverse DWT (IDWT),

(2) $$X_{0}={\mathrm{IDWT}}_{1D}\left(\mu \left({\mathrm{DWT}}_{1D}\left(X\right)\right)\right),$$

where $X_{0}\in R^{H\times W\times \frac{D}{4}}$, and μ(·) denotes a conv 1 × 1-BN-ReLU block that reduces the channel dimension.

To further capture joint spatial–frequency patterns, we apply a 2D DWT to $X_{0}$, decomposing it into four frequency subbands. These are then fused using a group convolution block φ(·) and reconstructed by 2D IDWT,

(3) $${\tilde{X}}_{0}=\varphi \left({\mathrm{DWT}}_{2D}\left(X_{0}\right)\right),\;X_{1}={\mathrm{IDWT}}_{2D}\left({\tilde{X}}_{0}\right),$$

where ${\tilde{X}}_{0}\in R^{\frac{H}{2}\times \frac{W}{2}\times D}$, $X_{1}\in R^{H\times W\times \frac{D}{4}}$, and μ(·) is implemented as a conv 3 × 3-BN-ReLU block. Note that ${\tilde{X}}_{0}$ is also shared with the WMSA branch for attention computation.

To reinforce directional structure modeling and enhance discriminative capability, we finally apply a 1D DWT to decompose the signal into directional low-frequency and high-frequency components again. The transformed representation is then compressed by a group convolution block μ(·) and reconstructed using 1D IDWT,

(4) $$X_{2}={\mathrm{IDWT}}_{1D}\left(\mu \left({\mathrm{DWT}}_{1D}\left(X_{1}\right)\right)\right),$$

where $X_{2}\in R^{H\times W\times \frac{D}{4}}$, and μ(·) denotes a conv 1 × 1-BN-ReLU block that restores the dimension of the channel.

Discussion. Compared with traditional bottlenecks, convolutions in the wavelet domain benefit from inherently larger receptive fields due to the spatial downsampling property of DWT. Specifically, since DWT reduces the spatial resolution by a factor of two, a standard 3×3 convolution applied in the wavelet-transformed space corresponds to an effective 6×6 receptive field in the original image space. This enables the model to aggregate broader contextual information at significantly lower computational cost, enhancing its ability to model long-range dependencies without increasing parameter count.

**WMSA.** To exploit the complementary nature of low-frequency and high-frequency components in the wavelet domain, we propose a wave fusion module (WFM) that selectively enhances structural representations using directional detail signals. This module splits the input $\tilde{X_{0}}\in R^{\frac{D}{2}\times \frac{W}{2}\times D}$ on the four subbands obtained from WBS: the approximation coefficients $X_{ll}$ and the detail coefficients $X_{lh},X_{hl},X_{hh}$ corresponding to horizontal, vertical, and diagonal orientations.

Instead of directly concatenating all four subbands, which may lead to feature redundancy or misalignment in importance, we adopt a residual-style enhancement strategy centered on the low-frequency base $X_{ll}$. Specifically, we treat the high-frequency responses as residual corrections to the coarse low-frequency map. The absolute values of the high-frequency subbands are used to emphasize edge and texture information while preserving the semantic context carried by $X_{ll}$,

(5) $$\begin{array}{cc} \; & X_{ll},X_{lh},X_{hl},X_{hh}=\mathrm{Split}\left({\tilde{X}}_{0}\right),\\ \; & {\tilde{X}}_{lh}=X_{ll}+|X_{lh}|,\;{\tilde{X}}_{hl}=X_{ll}+|X_{hl}|,\;{\tilde{X}}_{hh}=X_{ll}+\left|X_{hh}\right|,\\ \; & {\tilde{X}}_{1}=\mathrm{Concat}\left(X_{ll},{\tilde{X}}_{lh},{\tilde{X}}_{hl},{\tilde{X}}_{hh}\right), \end{array}$$

This fusion method is motivated by the observation that low-frequency wavelet coefficients preserve global structure and semantic content, while high-frequency components capture local discontinuities, such as edges and textures. However, directly using high-frequency maps as standalone inputs may amplify noise and background clutter. To mitigate this, we treat their absolute activations as refinement terms, aligning them with the low-frequency representation. Mathematically, this fusion can be interpreted as introducing anisotropic feature enhancement: directional derivatives in the wavelet domain serve as informative perturbations to a coarse base map. The result, ${\tilde{X}}_{1}\in R^{\frac{H}{2}\times \frac{W}{2}\times D}$, is thus a structurally enhanced frequency-aware feature map that balances locality and semantics.

We linearly project ${\tilde{X}}_{1}\in R^{\frac{H}{2}\times \frac{W}{2}\times D}$ to produce key $K^{w}$ and value $V^{w}$ embeddings for wavelet-enhanced multi-head self-attention (WMSA), allowing the attention mechanism to attend over both coarse and fine-grained frequency cues. The attention output per head is

(6) $$h_{j}=\mathrm{Softmax}\left(\frac{Q_{j}{\left(K_{j}^{w}\right)}^{⊤}}{\sqrt{D_{h}}}\right)V_{j}^{w},$$

where $Q_{j}\in R^{H\times W\times D}$, $K_{j}^{w},V_{j}^{w}\in R^{\frac{H}{2}\times \frac{W}{2}\times D}$. Before the dot product, $Q_{j}$ and $K_{j}^{w}$ are flattened along the spatial dimensions, resulting in $Q_{j}\in R^{(H\cdot W)\times D}$ and $K_{j}^{w}\in R^{(\frac{H}{2}\cdot \frac{W}{2})\times D}$. This produces an attention map of size $\left(H\cdot W\right)\times \left(\frac{H}{2}\cdot \frac{W}{2}\right)$, enabling cross-resolution attention where each high-resolution query attends to all coarse-scale key positions without explicitly downsampling $Q_{j}$.

**WMSA–WBS.** All attention heads are concatenated with the residual local feature $X_{\mathrm{WBS}}$ and projected

(7) $$\mathrm{WMSA}-\mathrm{WBS}\left(Q,K,V,X_{\mathrm{WBS}}\right)=\mathrm{Concat}\left(h_{1},h_{2}\ldots ,h_{n},X_{\mathrm{WBS}}\right)\cdot W,$$

where *W* is a learnable linear projection.

Complexity Analysis. Traditional MSA has $O(H^{2}W^{2}D)$ complexity. In WMSA–WBS, since key and value are computed from downsampled ${\tilde{X}}_{1}$, the attention cost is reduced to $O\left(\frac{H^{2}W^{2}D}{4}\right)$. The DWT/IDWT cost is linear, i.e., O(HWD), yielding an efficient design suitable for high-resolution vision tasks.

Comparison with Prior Wavelet Methods. While prior works have explored the integration of wavelet transforms into neural architectures, such as DWT-UNet [39] for segmentation and Wave-ViT [14] for vision transformers, they typically utilize wavelet decomposition as a preprocessing step or pooling replacement. In contrast, our proposed WBS introduces a residual wavelet bottleneck that leverages both 1D and 2D DWT-IDWT pipelines, enabling frequency-aware feature compression, fusion, and restoration within the network body. Moreover, unlike FFCNet [40] or WaveNet [41], which focus on global frequency aggregation or 1D dilation, WBS emphasizes spatial–frequency disentanglement and preserves the structural integrity of features via invertible transforms. These design choices allow WBS to be seamlessly integrated into transformer blocks (as in WMSA–WBS), achieving both contextual efficiency and spatial fidelity.

### 3.3. WMSA–WBS–ViT

**Figure 3:**
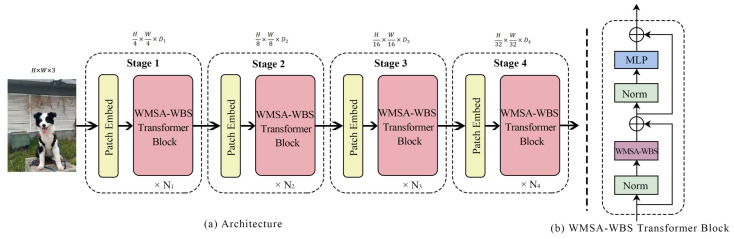
(**a**) The architecture of WMSA–WBS–ViT; (**b**) WMSA–WBS transformer blocks include WMSA–WBS and MLP module.

The overall architecture of the proposed WMSA–WBS vision transformer (WMSA–WBS–ViT) is illustrated in Figure 3a, while the internal structure of a single WMSA–WBS transformer block is shown in Figure 3b. Following the multi-scale vision transformer paradigm, we develop three model variants—WMSA–WBS–ViT-S, WMSA–WBS–ViT-B, and WMSA–WBS–ViT-L—differing in depth, width, and number of attention heads.

WMSA–WBS–ViT begins with a patch embedding layer, which partitions the input image $X\in R^{H\times W\times 3}$ into non-overlapping patches and projects them into an embedding space using a convolutional projection. This operation reduces the spatial resolution by a factor of 4 and projects each patch into a $D_{1}$-dimensional embedding, producing the Stage 1 feature map of size $\frac{H}{4}\times \frac{W}{4}\times D_{1}$. Compared with standard ViTs that apply linear patch flattening, our convolution-based embedding retains local spatial correlations and seamlessly integrates with hierarchical architectures. Subsequent stages further reduce the spatial resolution by a factor of 2 and increase the channel dimension, generating feature maps of sizes $\frac{H}{8}\times \frac{W}{8}\times D_{2}$, $\frac{H}{16}\times \frac{W}{16}\times D_{3}$, and $\frac{H}{32}\times \frac{W}{32}\times D_{4}$ at Stages 2 to 4, respectively. Each stage contains $N_{s}$ WMSA–WBS transformer blocks to progressively enrich the hierarchical representations. Each WMSA–WBS transformer block, as illustrated in Figure 3b, consists of two main components: the proposed WMSA–WBS module and a two-layer feed-forward MLP. Each component is preceded by a LayerNorm layer and followed by a residual connection. This design preserves the standard transformer structure while incorporating frequency-aware attention through WMSA–WBS. After Stage 4, the final feature map is globally pooled and fed into a classification head. Due to its modular and hierarchical design, WMSA–WBS–ViT can serve as a versatile backbone for various computer vision tasks. The detailed configurations of the three model variants are summarized in Table 1, where $E_{i}$, $H_{i}$, and $C_{i}$ denote the feed-forward expansion ratio, number of attention heads, and channel dimension in Stage *i*, respectively.

## 4. Experiments

In this section, we validate the performance of WMSA–WBS–ViT on a variety of vision tasks, including image classification on ImageNet1K, target detection and instance segmentation on COCO 2017, and semantic segmentation on ADE20K. Additionally, we perform ablation studies to validate the importance of key components in WMSA–WBS.

### 4.1. Image Classification

**Datasets.** Image classification experiments are conducted on the ImageNet-1K dataset [42], which is a subset of the larger ImageNet dataset and includes 1 K categories. The training set consists of 1.28 M images, while the validation set contains 50 K images, each sized at 224 × 224 pixels.

**Implementation Details.** All our vision backbones are trained from scratch using the training set. We evaluate the backbones on the validation set using Top-1 and Top-5 accuracy metrics. All experiments are conducted using the PyTorch 1.10.0 framework and accelerated by NVIDIA Quadro RTX 8000 GPUs. During training, we utilize AdamW [43] as the optimizer with a momentum of 0.9. We employ a cosine annealing schedule to adjust the learning rate, setting it to 0.001 and the weight decay to 0.05. The training lasts for 500 epochs, with a batch size of 1024 distributed across 8 GPUs.

**Results.**Table 2 presents a comparison between our proposed WMSA–WBS–ViT model and other mainstream backbone networks, including both transformer-based and ConvNet-based models, all trained on the ImageNet-1K dataset. Compared to the previous state-of-the-art transformer architectures, such as Swin transformer [44] and PVT [13], our proposed WMSA–WBS–ViT achieves higher accuracy while maintaining lower parameter counts and computational complexity. Specifically, WMSA–WBS–ViT-S (84.6%) improves by 3.3% over Swin-T (81.3%), while WMSA–WBS–ViT-B (85.3%) and WMSA–WBS–ViT-L (85.9%) exhibit significant performance advantages within their respective categories. Compared to ResNet, the WMSA–WBS–ViT series not only surpasses the ResNet series in terms of accuracy—WMSA–WBS–ViT-S, for example, improves by 7.4% over ResNet50—but also demonstrates clear advantages in computational efficiency. While these transformer models typically require a high computational burden, WMSA–WBS–ViT successfully controls the computational cost at a lower level through its efficient WMSA–WBS mechanism, and it holds great potential for further scalability.

### 4.2. Object Detection

**Datasets.** The COCO dataset [50] is a widely recognized benchmark in computer vision. We performed object detection experiments on this challenging benchmark. The COCO 2017 dataset includes 118 K training images and 5 K validation images. All models are trained on the COCO 2017 training set and evaluated on the COCO 2017 validation set.

**Implementation Details.** We use mainstream detector models in the MMDetection framework. We validate the effectiveness of the WMSA–WBS–ViT backbone using state-of-the-art detectors, including Cascade Mask R-CNN [51], ATSS [52], and Sparse R-CNN [53]. During training, images are resized so that the shorter side is fixed at 800 pixels, while the longer side does not exceed 1333 pixels. We use the SGD [54] optimizer with a learning rate of 0.01 and momentum of 0.9 to accelerate convergence. The batch size is configured to 16, and all models are trained using a 1× schedule. In the context of downstream object detection tasks, we present the average precision (AP) across a range of intersection over union (IoU) thresholds and for three object sizes: small, medium, and large (S/M/L).

**Results.** Our WMSA–WBS–ViT architecture demonstrates significant improvements across all the tested methods when compared to the ResNet-50 backbone in Table 3. Specifically, WMSA–WBS–ViT achieves consistent box AP gains ranging from 3.5 to 6.4 points, highlighting its superior performance in object detection tasks. Despite these gains, the increase in model size, FLOP, and latency remains modest. For semantic segmentation, in the Cascade Mask R-CNN framework, WMSA–WBS–ViT outperforms ResNet-50 by 4.9 box AP points while maintaining a comparable parameter count and a manageable increase in FLOP (from 1804 G to 2234 G). This trend is consistent across other methods like ATTS and Sparse R-CNN, where WMSA–WBS–ViT consistently outperforms ResNet-50 with only slight increases in computational complexity. We also compared the performance of different backbones. Specifically, we compared WMSA–WBS–ViT with three different backbones of Cascade Mask R-CNN. From Table 4, it can be concluded that WMSA–WBS–ViT improved performance by 2.2 to 3.9 points compared to the other three backbones, although its GFLOPs are slightly larger.

### 4.3. Semantic Segmentation

**Datasets.** ADE20K [56] is a commonly used semantic segmentation dataset featuring 150 categories, encompassing a range of objects and backgrounds in indoor and outdoor settings. It contains 25 K images in total, with 20 K for training, 2 K for validation, and 3 K for testing. We assess the pretrained WMSA–WBS–ViT on the semantic segmentation task using the ADE20K dataset.

**Implementation Details.** We utilize the MMSegmentation framework to improve the training efficiency by setting WMSA–WBS–ViT as the backbone network based on Semantic FPN [57] and UperNet [58]. We use fpnhead [20] as decode head for converting feature maps into segmentation masks. We also compare the segmentation performance with different backbone structures, including ResNet [27], ViT [1], Swin-ViT [44], PVT [13], and PVT V2 [12]. The images are first normalized and then cropped to 512 × 512. For data augmentation, we use the default MMSegmentation settings, which include random cropping, random horizontal flipping, and random photometric distortion. During training, an SGD optimizer is employed with a learning rate of 0.01 and momentum of 0.9 to enhance convergence. Additionally, a polynomial decay strategy is combined with a linear warm-up. The model is trained on eight GPUs, processing two images per GPU, for a total of 160 K iterations. After training, the mean Intersection over Union (mIoU) is utilized to evaluate the model’s average segmentation accuracy across categories.

**Figure 4:**
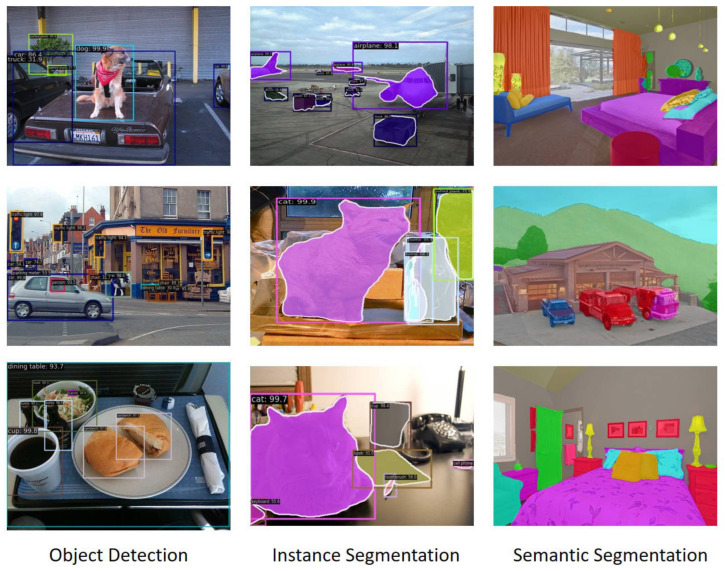
Results for object detection, instance segmentation, and semantic segmentation.

**Results.** The WMSA–WBS–ViT-S backbone demonstrates clear advantages in semantic segmentation tasks across different methods in Table 5. When integrated with Semantic FPN, WMSA–WBS–ViT-S not only achieves the highest mIoU of 47.2% but also does so with the lowest parameter count (23.4 M) and competitive FLOPs (40.4 G). The result indicates its efficiency and effectiveness over other backbones like ResNet50 and PVT versions. In the UperNet framework, WMSA–WBS–ViT-S continues to excel, achieving the highest mIoU of 49.3%, surpassing even the ViT-B16 backbone. Despite having a significantly lower parameter count (48.6 M) and only a slight increase in FLOP (240 G) compared to other models, WMSA–WBS–ViT-S offers the optimal balance between performance and computational cost. Figure 4 displays visualization results for object detection, instance segmentation on the COCO 2017 validation set, and semantic segmentation on ADE20K using the method presented in this paper.

The proposed WMSA–WBS–ViT has been evaluated across multiple downstream vision tasks, including image classification, object detection, and semantic segmentation, using the same ImageNet-pretrained backbone initialization. The strong performance achieved on COCO and ADE20K without substantial architectural changes or large-scale retraining suggests that the learned frequency-aware representations are transferable across tasks. This indicates that the proposed approach can be applied to other vision problems with minimal task-specific adaptation.

### 4.4. Ablation Study

We perform a series of ablation experiments to thoroughly examine the effect of various information types on the proposed WMSA–WBS–ViT.

**WBS.** WBS can be independently integrated within a CNN. We evaluate the impact of the WBS on the parameter count and Top-1 ACC of ResNet models through ablation experiments. Firstly, we assess the Top-1 ACC of the models on different datasets in Table 6. Table 7 shows a comparison of the parameter count between ResNet models with WBS and those with the traditional bottleneck. It can be seen that, with the introduction of the WBS, the parameter counts of ResNet50, ResNet101, and ResNet152 are reduced by 10.6%, 21.1%, and 24.7%, respectively. Despite the reduction in parameter count, ResNet models with the WBS still maintain or approach the Top-1 ACC of the original models in most cases. The results show that the WBS can reduce model complexity while preserving performance.

**Wavelet Types.** To further assess how different wavelet types affect the WBS, we compare the performance of various wavelet types on the Mini-ImageNet dataset. Table 8 presents the Top-1 and Top-5 ACC of the ResNet101 model using different wavelet types. In this experiment, we select seven wavelet types: haar, bior2.2, bior3.3, bior4.4, db2, db3, and db4. The experimental results show that different wavelet types have varying impacts on model performance. Among them, the model using db4 wavelet achieves the highest Top-1 ACC of 86.7% and a Top-5 ACC of 96.3%. This is followed by the db2 wavelet, with a Top-1 ACC of 86.5% and a Top-5 ACC of 96.3%. Other wavelet types, such as haar and bior2.2, performed slightly worse but still maintained high accuracy. These results indicate that selecting an appropriate wavelet type can enhance model performance to some extent. Specifically, the db4 wavelet performed the best in this experiment, possibly because it can more effectively capture image features, thereby improving the model’s classification ability.

**Figure 5:**
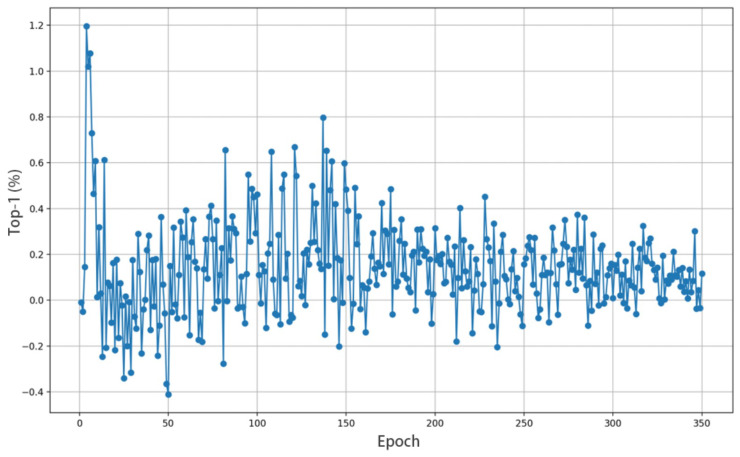
The difference in Top-1 ACC before and after removing high-frequency information using the WMSA–WBS.

**WMSA–WBS.** To validate the impact of high-frequency information on the WMSA–WBS, we conduct a differential analysis of Top-1 ACC. Specifically, we utilize the WMSA–WBS module to remove high-frequency information, retaining only low-frequency information, and then compare the resulting accuracy with that of the WMSA–WBS that incorporates both types of information. Figure 5 shows the difference in Top-1 ACC before and after removing high-frequency information using the WMSA–WBS. In this experiment, we calculate the changes in the model’s Top-1 ACC over different training epochs. As shown in the figure, in most cases, the majority of the differences are positive. The results show that the Top-1 ACC of WMSA–WBS, which preserves both high-frequency and low-frequency information, is considerably higher than that of the model that retains only low-frequency information. This further validates the beneficial influence of high-frequency information on enhancing model accuracy.

**Comparison with Different Self-Attention Models.** We compare WMSA–WBS with other multi-head self-attention variants to better validate WMSA–WBS performance. Specifically, traditional self-attention [59] serves as the foundation of attention mechanisms and is used as a baseline model for comparison. To address the reduction in computational complexity, efficient attention [24] and SRA [12] are selected for comparison. Efficient attention leverages sparsification and low-rank decomposition to effectively reduce parameter count and enhance computational efficiency. SRA, on the other hand, optimizes the attention mechanism by reducing the dimensions of key and value, further lowering computational costs. In terms of performance improvement, FET attention [22] and cross-attention [60] are chosen for comparison. FET attention introduces a novel attention computation method aimed at enhancing task-specific performance. Cross-attention improves model performance by integrating information from multiple feature spaces. We perform image classification experiments using the Mini-ImageNet dataset. From Table 9, it can be concluded that WMSA–WBS significantly outperforms the other multi-head self-attention variant.

**Figure 6:**
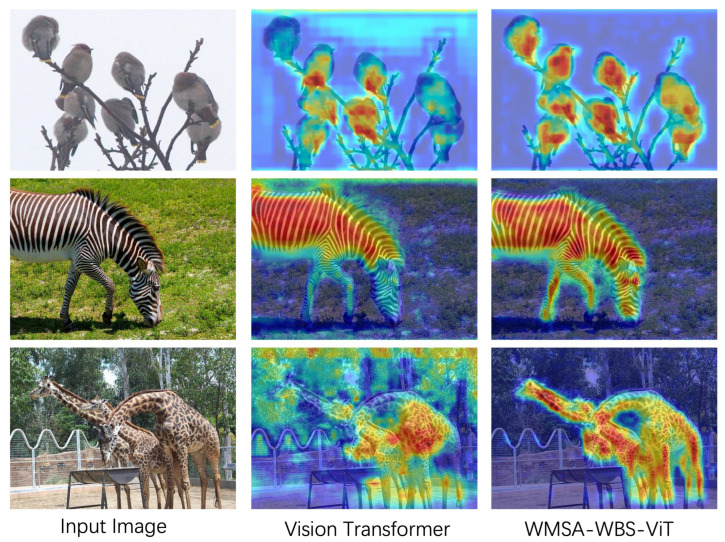
Grad-CAM visualization results.

**Visual Interpretability.** To further investigate the contribution of the proposed WMSA–WBS design to visual representation learning, we conduct a Grad-CAM-based [61] interpretability analysis. Figure 6 compares the activation maps of a baseline vision transformer and our WMSA–WBS–ViT on representative samples. The ViT shows dispersed and incomplete attention, whereas WMSA–WBS–ViT generates activation maps that align more closely with the full semantic regions of the target objects. This improvement is attributed to the frequency-aware feature modeling of WBS, which retains high-frequency edge and texture information, and the wavelet fusion in WMSA, which integrates both low- and high-frequency cues in the attention computation. These results indicate that WMSA–WBS–ViT enhances not only quantitative performance but also the interpretability and localization capability of ViT-based backbones, effectively attending to both global semantic regions and critical local details.

**Comparison with Different Downsampling Techniques.** To compare the effects of different downsampling operations on input key/value, we design experiments to evaluate their effects. From Table 10, we compare the downsampling effects of the max pooling operation, average pooling operation, and WMSA–WBS on the Mini-ImageNet dataset. Specifically, we first apply average pooling directly to the input key/value, achieving a Top-1 ACC of 88.5%. Next, we perform downsampling using max pooling, which results in a Top-1 ACC of 88.6%. Finally, using WMSA–WBS for downsampling achieved a Top-1 ACC of 89.2%, outperforming both pooling operations and further demonstrating that WMSA–WBS reduces computational costs while maintaining superior performance.

**WBS and WFM.** To evaluate the effectiveness of the proposed components, we conduct an ablation study on the wave bottleneck structure (WBS) and the wave fusion module (WFM). Table 11 reports Top-1 accuracy and parameter counts for different configurations. The baseline model without either module achieves 86.3% Top-1 accuracy. Introducing WBS alone improves accuracy by 2.5%, while WFM alone yields a 2.3% gain, both with marginal increases in parameters. When combined, WBS and WFM achieve the best performance of 89.2%, indicating that WBS and WFM are complementary: WBS enhances local detail representation through frequency-aware bottlenecking, and WFM further strengthens feature discrimination by integrating high- and low-frequency cues in the attention mechanism.

**Comparison of Channel Dimension Reduction at Different Positions.** We compare the effects of channel dimension reduction at different positions. Specifically, the first conv 1 × 1 in the 1D wavelet domain is used for reducing channel dimensions. However, there are two different structural configurations: one reduces channel dimensions after the wave fusion module and the other reduces them before the conv 3 × 3 in the 2D wavelet domain. To evaluate which structure provides better performance, we conducted experiments on the Mini-ImageNet dataset. The results are shown in Table 12, indicating that reducing the channel dimensions before the conv 3 × 3 in the 2D wavelet domain slightly improves the model’s Top-1 ACC, achieving 89.2%, compared to 89.1% when the reduction is performed after the wave fusion module. Despite the subtle difference in performance, this suggests that reducing the channel dimensions before the conv 3 × 3 in the 2D wavelet domain can enhance the model’s performance.

## 5. Conclusions

In this work, we propose the wave bottleneck structure (WBS), which extends the traditional bottleneck architecture by incorporating wavelet-based principles, thereby reducing the computational complexity of ResNet while preserving its accuracy. Building on this improvement, we introduce the WMSA–WBS module, which combines wave multi-head self-attention (WMSA) with WBS to enhance the performance of traditional MSA through the integration of high-frequency and low-frequency components derived from wavelet transforms. This design enables the module to capture detailed information across the spatial, frequency, and channel domains, encompassing both global and local contexts. To validate the proposed approach, we develop a series of WMSA–WBS–ViT models at different scales. In image recognition tasks, experiments show that WMSA–WBS–ViT outperforms state-of-the-art multi-scale ViT backbone networks while maintaining comparable parameter counts. Furthermore, the WMSA–WBS–ViT models demonstrate strong generalization, transferring effectively to downstream tasks such as object detection and semantic segmentation. While WMSA–WBS–ViT demonstrates notable improvements in accuracy, certain limitations remain. The module’s performance relies heavily on the complementarity between frequency-domain and spatial features, which may be less pronounced in scenarios dominated by low-frequency or low-texture content. Moreover, although its generalization capability has been validated on standard benchmark datasets, the method’s adaptability to cross-domain tasks—such as medical imaging and remote sensing—has not yet been comprehensively evaluated. Future work will explore applications in domains requiring detailed texture analysis, particularly concealed object detection, where wavelet transforms can capture fine-grained texture features.

## Figures and Tables

**Table 1:** The architecture specifications for the WMSA–WBS–ViT with three different model sizes, namely WMSA–WBS–ViT-S, WMSA–WBS–ViT-B, and WMSA–WBS–ViT-L.

	Output Size	WMSA–WBS–ViT-S	WMSA–WBS–ViT-B	WMSA–WBS–ViT-L
Stage 1	$\frac{H}{4}\times \frac{W}{4}$	$\left[\begin{array}{c} E_{1}=8\\ H_{1}=2\\ C_{1}=64 \end{array}\right]\times 3$	$\left[\begin{array}{c} E_{1}=8\\ H_{1}=2\\ C_{1}=64 \end{array}\right]\times 3$	$\left[\begin{array}{c} E_{1}=8\\ H_{1}=3\\ C_{1}=96 \end{array}\right]\times 3$
Stage 2	$\frac{H}{8}\times \frac{W}{8}$	$\left[\begin{array}{c} E_{2}=8\\ H_{2}=2\\ C_{2}=128 \end{array}\right]\times 4$	$\left[\begin{array}{c} E_{2}=8\\ H_{2}=4\\ C_{2}=128 \end{array}\right]\times 4$	$\left[\begin{array}{c} E_{2}=8\\ H_{2}=6\\ C_{2}=192 \end{array}\right]\times 6$
Stage 3	$\frac{H}{16}\times \frac{W}{16}$	$\left[\begin{array}{c} E_{3}=4\\ H_{3}=10\\ C_{3}=320 \end{array}\right]\times 6$	$\left[\begin{array}{c} E_{3}=4\\ H_{3}=10\\ C_{3}=320 \end{array}\right]\times 12$	$\left[\begin{array}{c} E_{3}=4\\ H_{3}=12\\ C_{3}=384 \end{array}\right]\times 18$
Stage 4	$\frac{H}{32}\times \frac{W}{32}$	$\left[\begin{array}{c} E_{4}=4\\ H_{4}=14\\ C_{4}=448 \end{array}\right]\times 3$	$\left[\begin{array}{c} E_{4}=4\\ H_{4}=16\\ C_{4}=512 \end{array}\right]\times 3$	$\left[\begin{array}{c} E_{4}=4\\ H_{4}=16\\ C_{4}=512 \end{array}\right]\times 3$

**Table 2:** Performance of various vision backbones on the ImageNet1K dataset.

Model	Parameter (M)	FLOP (G)	Top-1 ACC (%)
ResNet50 [27]	25.6	4.1	77.2
PVT-S [13]	24.5	3.8	79.8
MobileViT-XS [45]	2.3	0.7	74.8
EfficientFormer-L1 [46]	12.3	1.3	80.2
PVT v2-B2 [12]	25.4	4.0	82.1
NAT-T [47]	28.0	4.3	83.2
Swin-T [44]	29.0	4.5	81.3
VMamba-T [48]	30.0	4.9	82.6
Wave-ViT-S [14]	23.7	4.7	83.9
FasterViT-1 [49]	53.4	5.3	83.2
WMSA–WBS–ViT-S	23.2	4.9	84.6
ResNet101 [27]	44.7	7.9	78.3
PVT-M [13]	44.2	6.7	81.2
EfficientFormer-L3 [46]	31.3	3.9	82.4
PVT v2-B3 [12]	45.2	6.9	83.2
NAT-S [47]	51.0	7.8	83.7
Swin-S [44]	50.0	8.7	83.0
VMamba-S [48]	50.0	8.7	83.6
Wave-ViT-B [14]	33.5	7.2	84.8
FasterViT-2 [49]	75.9	8.7	84.2
WMSA–WBS–ViT-B	32.9	6.7	85.3
ResNet152 [27]	60.2	11.6	78.5
PVT-L [13]	61.4	9.8	81.7
EfficientFormer-L7 [46]	82.1	10.2	83.3
PVT v2-B4 [12]	62.6	10.1	83.6
NAT-B [47]	90.0	13.7	84.3
Swin-B [44]	88.0	15.4	83.5
VMamba-B [48]	89.0	15.4	83.9
Wave-ViT-L [14]	57.5	14.8	85.5
FasterViT-3 [49]	159.5	18.2	84.9
WMSA–WBS–ViT-L	55.7	13.8	85.9

**Table 3:** Object detection task on COCO dataset.

Method	Backbone	AP^box^	${\mathrm{AP}}_{50}^{\mathrm{box}}$	${\mathrm{AP}}_{75}^{\mathrm{box}}$	${\mathrm{AP}}_{S}$	${\mathrm{AP}}_{M}$	${\mathrm{AP}}_{L}$	Parameter (M)	FLOP (G)
Cascade Mask R-CNN [51]	ResNet-50 [27]	45.4	64.1	49.2	28.3	49.1	58.8	77.3	1804
	WMSA–WBS–ViT-S	50.3	70.3	55.3	31.7	50.6	59.9	77.3	2234
	WMSA–WBS–ViT-B	52.2	72.3	56.4	33.5	52.3	61.1	77.6	2314
	WMSA–WBS–ViT-L	53.3	73.2	57.3	34.2	52.6	62.2	77.9	2354
ATTS [52]	ResNet-50 [27]	39.4	57.6	42.8	23.6	42.9	50.3	32.3	184
	WMSA–WBS–ViT-S	45.4	66.5	50.4	29.2	50.2	59.5	32.1	195
	WMSA–WBS–ViT-B	47.4	66.7	52.4	31.2	52.2	61.5	32.4	201
	WMSA–WBS–ViT-L	48.5	67.3	53.5	32.5	53.4	62.2	32.7	213
Sparse R-CNN [53]	ResNet-50 [27]	42.8	61.7	46.2	26.2	44.9	58.2	106.0	136
	WMSA–WBS–ViT-S	45.3	67.1	50.6	30.2	51.2	60.5	106.2	159
	WMSA–WBS–ViT-B	47.3	69.1	52.6	32.2	53.2	62.5	106.3	159
	WMSA–WBS–ViT-L	48.3	69.9	53.6	33.2	54.2	63.5	106.9	159
Deformable-DETR [55]	ResNet-50 [27]	46.9	65.6	51.0	29.6	50.1	61.6	40.1	173
	WMSA–WBS–ViT-S	48.6	67.4	52.7	31.0	52.0	63.7	40.0	179
	WMSA–WBS–ViT-B	49.1	68.3	53.3	32.7	53.6	63.9	77.3	2234
	WMSA–WBS–ViT-L	50.3	70.3	55.3	33.4	54.3	64.3	77.3	2234

**Table 4:** Performance comparison of different backbone models in Cascade Mask R-CNN.

Backbone	${\mathrm{AP}}^{\mathrm{box}}$	${\mathrm{AP}}_{50}^{\mathrm{box}}$	${\mathrm{AP}}_{75}^{\mathrm{box}}$	${\mathrm{AP}}^{\mathrm{mask}}$	${\mathrm{AP}}_{50}^{\mathrm{mask}}$	${\mathrm{AP}}_{75}^{\mathrm{mask}}$	Parameter (M)	FLOP (G)
ResNet-50 [27]	46.4	64.1	50.2	40.2	61.5	43.1	77.3	1804
X101-32 [32]	44.3	62.7	48.4	38.3	59.7	41.2	101.5	1830
WMSA–WBS–ViT-S	50.3	70.3	55.3	42.3	67.2	48.8	87.3	2234
ResNet-101 [27]	46.4	64.1	50.2	40.2	61.5	43.1	77.3	1804
X101-64 [32]	45.3	63.9	49.6	39.2	61.1	42.2	101.5	1830
WMSA–WBS–ViT-B	52.2	72.3	56.4	45.2	67.2	48.9	87.0	2234

**Table 5:** Semantic segmentation results for different backbones.

Method	Backbone	Parameter (M)	FLOP (G)	MIoU (%)
Semantic FPN [57]	ResNet50 [27]	28.5	45.7	37.4
Semantic FPN [57]	PVT-Small [13]	28.2	44.5	39.8
Semantic FPN [57]	PVT V2-B2 [12]	26.3	41.0	45.2
Semantic FPN [57]	WMSA–WBS–ViT-S	23.4	**40.4**	**47.2**
UperNet [58]	ResNet50 [27]	64.1	**238**	40.7
UperNet[58]	Swin-ViT-T [44]	59.0	237	44.4
UperNet [58]	ViT-b16 [1]	142	444	47.7
UperNet [58]	WMSA–WBS–ViT-S	48.7	240	49.3

**Table 6:** Comparison of ResNet with wave bottleneck and ResNet regarding parameters and Top-1 ACC on different datasets.

Dataset	Model	Parameter (M)	Top-1 ACC (%)
CIFAR-10	ResNet50 [27]	25.5	97.1
	ResNet101 [27]	44.5	97.6
	ResNet152 [27]	60.1	97.9
	ResNet50 + WBS	22.8	96.6
	ResNet101 + WBS	35.1	97.3
	ResNet152 + WBS	45.3	97.5
CIFAR-100	ResNet50 [27]	25.5	83.4
	ResNet101 [27]	44.5	83.7
	ResNet152 [27]	60.1	83.5
	ResNet50 + WBS	22.8	82.4
	ResNet101 + WBS	35.1	83.5
	ResNet152 + WBS	45.3	83.6
Mini-ImageNet	ResNet50 [27]	25.5	86.9
	ResNet101 [27]	44.5	87.3
	ResNet152 [27]	60.1	87.5
	ResNet50 + WBS	22.8	85.9
	ResNet101 + WBS	35.1	86.7
	ResNet152 + WBS	45.3	86.8

**Table 7:** Reduction in parameter count of ResNet with WBS compared to ResNet with bottleneck.

Model	Bottleneck (M)	WBS (M)	Parameter Reduction
ResNet50 [27]	25.5	22.8	10.6% ↓
ResNet101 [27]	44.5	35.1	21.1% ↓
ResNet152 [27]	60.1	45.3	24.7% ↓

Note: The downward arrows (↓) indicate the percentage reduction in parameters compared to the bottleneck design.

**Table 8:** Comparison of ResNet101 using WBS on the Mini-ImageNet dataset using different wavelet types.

Wavelet Type	Top-1 ACC (%)	Top-5 ACC (%)
haar	86.3	96.3
bior2.2	86.3	96.3
bior3.3	86.2	96.1
bior4.4	86.2	96.2
db2	86.5	96.3
db3	86.4	96.3
db4	86.7	96.3

**Table 9:** Effects with different self-attention models.

Method	Parameter	Top-1 ACC (%)
Self-Attention	22.2	86.3
Efficient Attention	22.3	86.3
FET Attention	23.5	87.8
Cross-Attention	23.3	88.9
SRA	23.9	89.0
WMSA–WBS	23.2	89.2

**Table 10:** The effects of different downsampling operations on input key/value.

Method	Parameter	Top-1 ACC (%)
Avg-Pool	22.9	88.5
Max-Pool	22.9	88.6
Our method	23.2	89.2

**Table 11:** Performance comparison of WBS and wave fusion module.

WBS	WFM	Parameter	Top-1 ACC (%)
-	-	22.2	86.3
✓	-	23.2	88.8
-	✓	22.8	88.6
✓	✓	23.2	89.2

Note: ‘✓’ indicates that the corresponding module is used; ‘-’ indicates that it is not used.

**Table 12:** The effects of channel dimension reduction at different positions.

Method	Parameter (M)	Top-1 ACC (%)
After Wave Fusion Module	23.2	89.1
Before conv 3 × 3 in the 2D Wavelet Domain	23.2	89.2

## Data Availability

The data that support the findings of this study are openly available at https://cocodataset.org/ and https://image-net.org.
